# Brain anatomy alterations associated with Social Networking Site (SNS) addiction

**DOI:** 10.1038/srep45064

**Published:** 2017-03-23

**Authors:** Qinghua He, Ofir Turel, Antoine Bechara

**Affiliations:** 1 Faculty of Psychology, Southwest University, Beibei, Chongqing, China; 2 Brain and Creativity Institute, Department of Psychology, University of Southern California, Los Angeles, California, USA; 3 Information Systems and Decision Sciences, California State University, Fullerton, Fullerton, California, USA

## Abstract

This study relies on knowledge regarding the neuroplasticity of dual-system components that govern addiction and excessive behavior and suggests that alterations in the grey matter volumes, i.e., brain morphology, of specific regions of interest are associated with technology-related addictions. Using voxel based morphometry (VBM) applied to structural Magnetic Resonance Imaging (MRI) scans of twenty social network site (SNS) users with varying degrees of SNS addiction, we show that SNS addiction is associated with a presumably more efficient impulsive brain system, manifested through reduced grey matter volumes in the amygdala bilaterally (but not with structural differences in the Nucleus Accumbens). In this regard, SNS addiction is similar in terms of brain anatomy alterations to other (substance, gambling etc.) addictions. We also show that in contrast to other addictions in which the anterior-/ mid- cingulate cortex is impaired and fails to support the needed inhibition, which manifests through reduced grey matter volumes, this region is presumed to be healthy in our sample and its grey matter volume is positively correlated with one’s level of SNS addiction. These findings portray an anatomical morphology model of SNS addiction and point to brain morphology similarities and differences between technology addictions and substance and gambling addictions.

Notwithstanding the positive impacts of technologies on humans, technology-related addictions seem to be fairly prevalent^1^,^2^; A recent meta-analysis suggests that globally the prevalence rate is about 6% and that it varies by country, ranging from 2.6% to 10.9%^3^. While the negative outcomes of such addictions may not always be as devastating as those generated by severe substance addictions, they attack the vulnerable population of adolescents and young-adults^4^,^5^ and can have a myriad of negative effects on individuals’ work, school and social functioning, wellbeing and psychological states^2^, as well as on their sleep hygiene and long-term cardio-metabolic health^5^. Therefore, these addictions have been recognized as an important topic that merits further research^6^ and the fifth edition of diagnostic and statistical manual for mental disorders has included the concept of “Internet Gaming Disorder” in the appendix (section 3, potential disorders requiring further research)^7^. Conceptual psychological-neurobiological models^8^ as well as functional brain imaging studies^9^ suggest that such addictions involve *an interaction of sensitized reward processing and cue-reactivity with diminished prefrontal inhibitory control.* Yet, more research is needed for understanding the structural neural underpinnings of this phenomenon^10^. Specifically, even though addictions are recognized as “brain diseases” by the American Medical Association, little is known regarding potential brain structural alterations associated with such addictions; this knowledge can help researchers and medical practitioners develop interventions for preventing or treating such addictions.

As such, this study seeks to examine potential brain structural alterations associated with an important instance of technology addictions, namely addiction to a social networking site. Social Networking Site (SNS) addiction is a subcategory of the technology/Internet spectrum of addictions^11^ and is defined as a user’s maladaptive psychological state of dependency on the use of an SNS, which is manifested through an obsessive pattern of seeking and using this SNS such that these acts infringe normal functioning and produce a range of typical behavioral addiction symptoms, including salience, withdrawal, relapse, growing tolerance, conflict and mood modification^12^. While there is stronger consensus regarding the prevalence of maladaptive technology use patterns which result in addiction-like symptoms^1^,^2^, it is not clear yet if the term “addiction” is best, and whether other terms such as “use disorder” may be more appropriate. This study, however, uses the term “addiction” in line with prior research in this field, even though the medical community still debates if this term is appropriate^6^. Furthermore, in line with this line of work^13^ we treat addiction as a continuous concept, i.e., we capture the level of addiction-like symptoms all people have, rather than trying to medically classify people as addicts or non-addicts using non-established criteria.

This study specifically focuses on brain anatomy modulations in terms of the grey matter volumes (GMV; see glossary of neuroscience terms in Appendix A) of brain regions, which are arguably associated with SNS addiction and are flexible or prone to anatomical modulations. These alterations are presumed to take place in central and necessary regions of the dual-system which governs behavior^14^, the deficiency of which is associated with addictions^15^. These regions are: (1) the Nucleus Accumbens (NAc), which has been implicated in playing a primary role in addictive behaviors through the processing of rewards that motivate behavior, including problematic behaviors; (2) the amygdala, which has been implicated in playing a key role in triggering impulsive behaviors from conditioned cues; presumably by linking environmental cues to neural systems involved in negative reinforcement (e.g., the relief from an aversive condition such as withdrawal), as well as positive reward and reward expectancy, such as those mediated by the NAc^16^; and (3) the midcingulate cortex (MCC), i.e., the dorsal region of the anterior cingulate cortex (ACC), which is involved with self-control or inhibition processes in response to impulsions generated through the impulsive system. The glossary in Appendix A provides details regarding these neural substrates.

Addiction is often initiated by hyperactivity of the system that assesses rewards^17^ and drives impulsive behaviors^15^. This includes the NAc, the key substrate where mesolimbic dopamine is released, and reward seeking behavior is elicited, and it also includes the amygdala, which is thought to link environmental cues to reward systems in the striatum, including the NAc. This system can become over-sensitized through repetitive enactment of a rewarding behavior and recurring strong intrinsic rewards, which can lead to a constant state of “wanting” to enact the addictive behavior^18^. The NAc is a central and necessary component of this reward system^19^, but the amygdala has also been argued as a necessary component of a broader neural system underlying automatic, habit, and impulsive behaviors^15^,^20^,^21^. Hence, addictions are typically advanced by hyperactivity of the extended amygdala circuit which includes the NAc and amygdala^16^. Many subcortical reward-system regions^10^, as opposed to cortical regions, are morphologically flexible and can easily adjust to new environmental demands^22^. Hence, it is reasonable to assume that addiction-associated morphology changes (see glossary of neuroscience terms in Appendix A), if exist; can apply to the NAc and amygdala.

Oftentimes, the increased efficiency of the extended amygdala (reward) system is manifested through pruning wasteful and redundant neurons, and specifically reducing the GMV of the amygdala such that lean, fast and competent, bundles of neurons are retained. Achieving higher performance through pruning is very common^23^ and is especially relevant in subcortical areas^24^. It should be noted that while grey matter volume reduction changes to such regions are similar across addictions^19^,^20^, the processes that lead to such changes may differ between addictions. In many cases, substances such as cocaine, which bind to dopamine receptors, create direct neurobiological changes in the operation and GMV of such brain regions^25^. In behavioral addictions, in contrast, such as addiction to SNS or videogame use, the implicated systems are typically affected indirectly, by environmental behaviors^26^,^27^, through changing the work demands imposed on these brain regions, e.g., through increasing the need for reward or task-conflict processing and the resultant natural brain adaptations^28^.

Regardless of the process, negative associations between the GMV of the (typically bilateral) amygdala and other addictions have been observed in both substance and behavioral addictions, including for example in cases of abuse of cannabis^29^, alcohol^30^, cocaine^31^, prescription opioids^32^, as well as in problematic behaviors such as gambling^33^. Given possible neural and behavioral similarities between other addictions and technology-related addictions^34^, and the shared neural basis of different addictions^21^ including behavioral ones^27^, it is reasonable to expect that such negative associations also exist in the cases of SNS addiction. We hence hypothesize that (H1) the grey matter volume of the amygdala will be negatively associated with one’s SNS addiction score; after controlling for age, gender, number of contacts on the SNS, SNS use frequency, years of experience with the SNS, and the whole brain GMV. We suggest controlling for demographic and SNS use variables to ensure that the observed variation in GMV is associated with addiction per-se. We also suggest cleaning any variance in GMV which may be attributed to general brain volume of grey matter, across regions, which may differ from one individual to another and influence the GMV of the examined regions of interest (ROIs) regardless of addiction.

While the NAc is a central and active region in all addiction phases^16^, the existence and direction of possible structural differences in the NAc in relation to addictions are not clear. Some studies, for example, show GMV reduction in right NAc in alcoholism cases^30^ or left NAc in heroin-dependent patients^35^; whereas others show increased GMV of left NAc in cannabis users^29^ and frequent video-gamers^36^. Some studies, albeit focusing on connectivity, did not find correlations of NAc connectivity with sharing of self-related information on social media^37^. Given these mixed findings, and also the fact that the NAc is anatomically difficult to define with precision on scan images, we refrain from hypothesizing about the existence and direction of structural differences in the NAc. Nevertheless, given the centrality of the NAc in reward processing, including in the case of social media use^38^ we explore post-hoc whether structural differences in the NAc are associated with SNS addiction.

In addition to the abovementioned hyperactivity of the impulsive/reward assessment brain system, addictions typically also involve hypo-activity of the reflective or inhibition brain system^15^. This hypo-activity is often reflected in these areas of the brain through reduced grey matter^39^,^40^,^41^. The ACC/MCC is of particular interest since it is relevant for weak inhibition abilities and consequent addictions; and the grey matter morphology of the ACC/MCC has been linked to addictive and excessive behaviors^42^. In such cases the ACC/MCC changes typically manifest through reduced GMV of this region, e.g., among methamphetamine users^39^, excessive-eaters^40^, cocaine users^41^, and Internet addicts^43^. Hence, if SNS addiction is similar to other addictions, and given that addictions are largely similar in their neural roots^21^ it is reasonable to assume that it is negatively associated with the GMV of the ACC/MCC, possibly reflecting lower efficiency of this inhibition system region. We hence hypothesize that (H2) the grey matter volume of the ACC/MCC will be negatively associated with one’s SNS addiction score; after controlling for age, gender, number of contacts, SNS use frequency, years of experience with the SNS, and the whole brain GMV.

## Results

The addiction scale was statistically valid and reliable (α = 0.91, composite reliability = 0.93, and Average Variance Extracted = 0.63). Hence, its average represented participants’ presumed addiction levels or at least their levels of addiction-like symptoms. Addiction scores (average = 2.200, SD = 0.718, Range = 1.071 to 3.653 on a 1–5 Likert type scale) as well as all extracted GMVs did not deviate from normality (Kolmogorov-Smirnov and Shapiro-Wilk test statistics all with p-values > 0.20). Hence, correlational analyses were deemed to be appropriate. Addiction scores were not significantly correlated with age, years of Facebook experience, and number of contacts; but correlated with sex (r = 0.44, p < 0.05) and frequency of use (r = 0.74, p < 0.000). Hence, women in our sample had higher addiction scores than men had, and higher addiction scores were, as expected, associated with more frequent use of Facebook.

**Figure 1:**
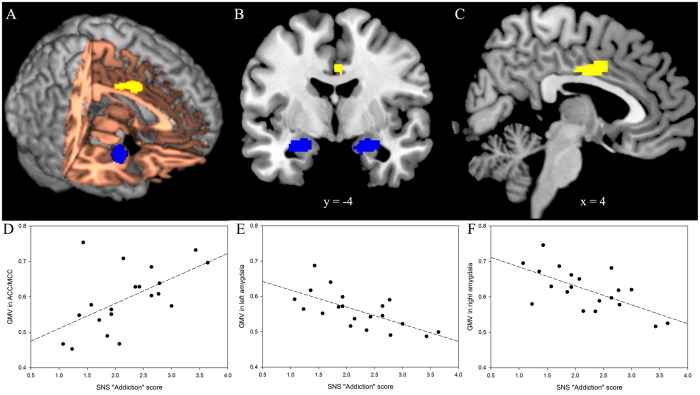
Visualization of the voxel-wise VBM results*. *Voxel-wise VBM results illustrated in three different views: rendered brain (**A**), coronal view (**B**), and sagittal view (**C**). The SNS addiction score was negatively correlated with GMV in bilateral amygdala (blue areas) and positively correlated with GMV in the anterior/mid cingulate cortex (ACC/MCC, yellow area). Slices are displayed in radiological view (right is on the viewer’s left). Scatter plots (**D–F**) show the pattern of correlation between GMV (**D)**: ACC/MCC; (**E**): left amygdala; (**F**): right amygdala) and SNS addiction score.

Voxel-wise based morphometry (VBM, see glossary in Appendix A) analyses revealed that SNS addiction scores, as hypothesized, negatively correlated with GMV in the bilateral amygdala (left amygdala, local maxima in MNI coordinates x, y, z = −30, −8, −18, geometric center x, y, z = −24.3, −5.6, −17.4; right amygdala, local maxima x, y, z = 30, 0, −14, geometric center x, y, z = 23.7, −3.5, −18.0), after accounting for control variable effects (see Table 1 and Fig. 1). Hence, H1 was supported. The VBM analyses, however, revealed, in contrast to our expectation, that the SNS addiction score was positively (and not negatively) correlated with GMV in the ACC/MCC (local maxima x, y, z = 4, −8, 34, geometric center x, y, z = 3.3, 0.2, 36.1), after accounting for control variables (see Table and Fig. 1). Hence, H2 was not supported. In addition, voxel-wise analysis did not show significant correlation between addiction scores and GMV in bilateral NAc.

To supplement the voxel-wise analysis, additional theory driven ROI analyses were performed. The average GMVs in five anatomically defined ROIs (bilateral amygdala, ACC/MCC, and bilateral NAc) were extracted and partially correlated with the addiction score. Results suggested that addiction scores were negatively correlated with the left and right amygdala volumes (r = −0.67, p < 0.01 and r = − 0.65, p < 0.01, respectively) and positively correlated with MCC volumes (r = 0.57, p < 0.01). Left and right NAc volumes did not significantly correlate with addiction scores (p < 0.52 and p < 0.76, respectively). In order to alleviate possible power concerns, the analyses were repeated without the control variables and produced similar results. In order to further examine the non-significant association of NAc volumes with addiction scores, cross-validation with 100 re-samples was performed. It produced non-significant confidence intervals for this association (Left: −0.587 to 0.340; right: −0.520 to 0.344). Thus, while it is possible that the non-significant NAc associations are due to low power, our findings indicate that there may also be other reasons for this.

## Discussion

This study sought to examine brain structural alterations associated with SNS addiction. Our findings lend support to the idea that the composition of key brain regions of the dual-system of reasoning, the amygdala and ACC/MCC, but not the NAc, is associated with SNS addiction. We specifically show that the GMV of the amygdala is negatively associated with SNS addiction scores. Hence, people with high SNS addiction scores have a pruned amygdala, which is presumably involved in generating strong impulsive behaviors. In this respect, SNS addiction is similar to other types of substance and behavioral addictions, in which case addicts present reduced GMV of the amygdala^29^,^30^,^32^,^33^. In contrast, the observed differences in the GMV of the ACC/MCC were opposite in direction to what we hypothesized based on accumulated knowledge regarding substance addiction and excessive eating. Research in the substance^39^,^41^ and food addiction^40^ domains typically shows that addicts have deficient ACC/MCC and lower decision conflict resolution abilities, which is manifested through reduced GMV. While similar observations have not been made in the case of most behavioral addictions, reduced GMV of cingulate gyri have been observed in impulsivity and anxiety disorders that do not involve substances, such as in the case of obsessive-compulsive disorder^44^. In this study, however, we found positive association between the GMV of the ACC/MCC and SNS addiction. In addition, the findings did not implicate structural changes in the NAc in SNS addiction, which is consistent with other studies on social media use^37^, but inconsistent with studies on substance addictions^29^,^30^. This suggests important potential differences between SNS addiction and commonly studied substance and behavioral addictions and points to the need to possibly consider treatments for technology addictions which differ from those offered for other addictions. Together, the findings of this study portray an anatomical morphology model of SNS addiction.

Several implications of the findings are noteworthy. First, the findings point to possible key differences between SNS addiction and substance addictions. A fundamental difference is that while addictions to illicit substances are often associated with decreased grey matter in frontal cortical systems involved in decision-making and self-control^30^,^45^,^46^,^47^, SNS addiction seems to be associated with increased grey matter in these regions (at least the ACC/MCC). One potential explanation for this, which we cannot support or refute in this study, is that inter-individual differences in the GMV of the ACC/MCC may be a result of an adaptation and compensation processes, which is executed in response to the sub-cortical modulatory changes in the amygdala; this represents a “normal adaptation response” proposition that merits further research. That is, the ACC/MCC of users in our sample may be healthy (as opposed to the ACC/MCC in samples of substance addicts) and consequently, it adapts to the growing efficiency of the impulsive system by bundling neighboring neurons and growing new ones; i.e., through GMV increase. A similar argument can be applied to the NAc; it is possible that in rather healthy users it is not modulated, and that changes in the amygdala alone, without further changes to the extended amygdala circuitry^16^, suffice for producing stronger motivation to use the SNS. Note that this does not imply that the NAc is less central than the amygdala in addiction development and maintenance; it only means that all brain regions are potentially different in terms of their sensitivity to structural changes incurred by the SNS behavior under study.

In indirect support of this *normal adaptation response* proposition, it has been shown that in healthy subjects, impulsivity is associated with an increase in the GMV of the ACC/MCC^48^. Further indirect support to this explanation is provided by showing that working memory for Internet-words was improved rather than worsened (as hypothesized) among Internet addicts^49^. In both cases the unexpected positive changes, like in our case, may represent a healthy and normal adaptation to the increase in impulsions, which may take place among people with not too high impulsivity and addiction scores (i.e., who can be classified as rather cognitively healthy). This may not be the case for people with very high addiction and impulsivity scores, who are largely excluded from all of the abovementioned samples, including ours (Note that subjects in our sample presented no clinical impairments on the Structured Clinical Interview for DSM-IV (the SCID) and hence can be viewed as rather cognitively and mentally healthy). The *normal adaptation response* proposition we describe here, however, merits further exploration and provides an interesting springboard for future research.

Second, the findings reinforce the well-established dual-system theory and conceptual psychological-neuroscientific models of Internet addiction, such as I-PACE^8^ using a brain morphology angle. While much research has focused on the behaviors and brain activations associated with the dual-system that governs behavior^15^, and models such as I-PACE suggest that Internet addiction is a function of an interaction of limbic and prefrontal brain areas^8^, this study reinforces the anatomical-morphologic basis of dual-system dysfunction which underlies technology addictions. Our findings are consistent with past research showing that deficiencies of this dual system or parts of it are perhaps rooted in the neuroplasticity of several brain regions of this system^41^,^50^. They further emphasize the relevance of dual-system theories and the I-PACE model for research on problematic online behaviors. We hence call for further use of this theory; both in behavioral and neuroscience information systems use research.

Third, while this study points to important associations and insights, it is still premature for us to use such insights for making definitive practical suggestions. As such, our recommendations involve further research based on the directions set by our findings. Specifically, it is possible to reverse structural morphology changes in the brain; i.e., apply neuroplasticity targeted at positive changes, through behavior change interventions^51^, practicing mindfulness^52^, the use of noninvasive Transcranial Magnetic Stimulation or its repeated administration version (TMS and rTMS respectively)^53^, or pharmacological drugs that promote neurogenesis^54^. All of these techniques have demonstrated potential to alter brain microstructures such as grey matter. They may work well in the case of non-substance addictions such as SNS addiction, because neurotoxicity is not involved in such cases (as opposed to cases of substance addiction in which neurotoxicity may prevent recovery of some of the structural changes in the brain). Nevertheless, the usefulness of such techniques for dealing with the specific changes required for treating SNS addiction is unknown and should be further studied.

Several limitations of this study should be acknowledged as they point to future research directions. First, the power of our analysis could not be determined with existing VBM tools. It should therefore be assumed that we did not have high power and that our sample may have resulted in both Type I and Type II errors. This suggests that the non-significant findings may be an artifact as well as that the significant results may have been inflated. Hence caution should be exercised when interpreting the results and replication of our results with larger samples is highly desirable. Second, this study was conducted in a single context. The model should be replicated with other SNS and populations. Third, although participants with relevant psychiatric diagnosis were not found, it is possible that sub-clinical levels of psychiatric disorders could influence the findings. Future research can increase the range of potential confounds to be considered. Fourth, this study was correlational in nature. Even though there was a time-lag between the structural scans and survey completion, perfect causality cannot be demonstrated. Future research may employ longitudinal designs in order to be able to establish causality. Fifth, future research may examine why the changes in GMV of the ACC/MCC in the case of SNS addiction differ from those observed in the case of other addictions. Sixth, it would be desirable to examine how changes in GMV translate into changes in activity, and this can be done using fMRI and MRI techniques in tandem in future research. Lastly, while we associate grey matter volumes with one’s level of addiction, the techniques we use do not allow us to examine the different components of grey matter. These can include neuronal and glial cells, axon terminals, and dendrites. It is unclear how the composition of grey matter (amount of each one of these substructures, and the balance among these substructures) influence the computational efficiency of regions of interest^22^. Future research can use more microscopic techniques to examine such issues.

Overall, this study demonstrated that brain morphology could serve as a marker for one’s level of SNS addiction. It specifically shows that human-technology interactions may be linked to the grey matter composition of brain regions which govern behavior. The findings therefore contribute to the growing body of work on the neural basis of problematic technology, and specifically social media, use^10^. They also suggest that in the future we should consider finding interventions targeted at brain anatomy that may correct problematic technology use behaviors. Reflecting on how the tobacco industry suffered from not being proactive in its early years regarding trying to prevent harms to its customers, we hope that this study serves as a springboard for the research community to be more proactive regarding the understanding, detection, prevention and treatment of technology addictions and other “dark side” of technology use phenomena.

## Methods

### Recruitment protocol

The study protocol was approved by the institutional review boards of the University of Southern California and California State University, Fullerton. All methods were performed in accordance with the relevant guidelines and regulations. Fifty participants were recruited by means of advertising on a bulletin board and completed a screening survey and a computerized short version of the Structured Clinical Interview for DSM-IV (the SCID). Inclusion criteria were (1) over 18 years old, and (2) Facebook use. The exclusion criteria were existence of any (1) self-reported common medical conditions which may affect relevant brain regions, and (2) mental disorders that may confound our results. No exclusions were made. Out of the 50 survey participants, twenty were invited and agreed to be scanned with an MRI device a week later. Selection was based on addiction scores and sex. We wanted to ensure that the sample includes varying levels of addiction scores and equal proportions of sexes.

### Participants

The sample comprised of twenty Facebook users. Participants’ average age was 20.3 years (18 to 23; 10 women, 10 men). They had on average 4.7 years of Facebook experience (0.4 to 9), 743 Facebook contacts (130 to 2500), and used Facebook 8.4 times per day (1–25). Informed consent was obtained from all participants, they met the inclusion criteria and they did not meet any of the exclusion criteria. All of them were self-reportedly healthy (they may have had sub-clinical diagnoses that the SCID does not capture, though) and had no mental or medical issues that should preclude them from participation, which is expected given that the participants were presumably normal.

### Measures

Measures were obtained from three sources. First, participants were asked to complete an online survey that captured their levels of SNS addiction, demographic and descriptive variables, as well as inclusion-exclusion criteria items. Second, participants completed screening criteria forms for capturing medical issues that may put individuals at risk in the scanner as well as specific mental conditions and substance addictions that would exclude individuals from this study. These data were used for exclusion-inclusion decisions but not for hypothesis testing. Lastly, grey matter volumes of brain regions were captured by applying Voxel-Based Morphometry techniques to high-resolution structural MRI scans, which were conducted one week after the surveys were completed.

The online survey (see Appendix B) captured people’s age, number of contacts on the SNS, the frequency in which they use the SNS, and years of experience with the SNS using open-ended numeric questions. It also captured participants’ sex. The survey also screened for various medical conditions which may bias the results using self-reports. Lastly, the survey captured SNS addiction scores by using the 14-item instrument by Van Roij *et al*.^55^ as adapted from Meerkerk *et al*.^56^. This scale taps into typical behavioral addiction symptoms such as withdrawal, salience, relapse, loss of control, and conflict. The score it produces, therefore, captures people’s level of addiction as manifested by these symptoms. The original addiction instrument focused on videogames, and it was modified to focus on SNS by replacing videogames with SNS in the statements, and retaining all the symptoms the original scale captures. To ensure its validity in the new context it was first presented to four experts in behavioral addiction who confirmed its content validity and its ability to capture key aspects of all relevant symptoms. It was then pre-tested with a sample of 40 SNS (Facebook) users, and showed good psychometric properties (alpha = 0.90, composite reliability = 0.92, and Average Variance Extracted (AVE) = 0.58). Consequently, it deemed to be appropriate for our study.

After completing this survey, the screening forms captured more general inclusion-exclusion criteria, which have to do with participant safety, such as pregnancy, having pacemakers, neuro-stimulators, head trauma and asthma. Individuals were also screened for specific mental disorders and other addictions which would exclude them from this study using a short computerized version of the Structured Clinical Interview for DSM-IV (the SCID). We used a version that covers only presumably relevant disorders because it is shorter to administer^57^, as opposed to the full SCID^58^. This interview was used to assign Axis I diagnoses, looked at both lifetime and current diagnoses and was conducted by a trained research assistant who had several years of experience using this tool. It took about 20 minutes and focused on capturing specific relevant issues such as psychosis, depression, heavy drinking (abuse and dependence), substance abuse, pathological gambling, schizophrenia, anxiety disorders, or bipolar disorder which would not allow proper MRI scanning or present problematic comorbidities.

Lastly, high-resolution structural scans were used for performing morphological analysis, one week after the surveys were completed. Specifically, voxel-wise grey matter volumes were partially correlated with addiction score and other covariates. Significant findings from the voxel-wise GMV correlation were extracted for plotting purposes. The GMV of five anatomically defined hypothesis-driven ROIs (bilateral amygdala, bilateral NAc, and MCC) were also extracted to provide further support to the hypotheses; they were partially correlated with addiction scores.

### MRI Protocol and VBM Analysis

Magnetic Resonance Imaging (MRI) scans were performed in a 3 T Siemens MAGNETOM Tim/Trio scanner in the Imaging Center at University of Southern California. The MRI session lasted about 15 minutes, and one high-resolution structural scan was performed. The T1-weighted 3D-Magnetization Prepared RApid Gradient Echo (MPRAGE) sequence was used to cover the whole brain (TR (repetition time)/TE (echo time) = 2530/3.39 ms, flip angel = 7^o^, matrix = 256 × 256, 128 sagittal slices, 1.33 mm thickness). The scanned brains were divided into voxels (the three dimensional equivalent of pixels) with volumes of 1 mm^3^. These voxels served as the smallest data point for further analyses.

Because people have brains different in size and shape, pre-processing of brain images was performed to bring all brains into a common three-dimensional space, and voxels’ locations were mapped onto a common anatomical brain map. This preprocessing and consequent data analyses were done with FSL (FMRIB Software Library)-VBM (voxel-based morphometry) (http://fsl.fmrib.ox.ac.uk/fsl/fslwiki/FSLVBM/), an optimized voxel-based morphometry analysis toolbox implemented in FSL, a statistical toolbox for neuroimaging data (http://fsl.fmrib.ox.ac.uk/fsl/fslwiki/FSL). This approach has been proven to be efficient and operator-independent^59^. First, structural images were extracted using the Brain Extraction Tool, BET. Next, tissue-type segmentation was carried out using the FMRIB’s Automated Segmentation Tool, FAST4. The resulting grey matter partial volume images were then aligned to the grey matter template in the Montreal Neurological Institute standard space (MNI152) using the affine registration tool FLIRT (FMRIB’s Linear Image Registration Tool), followed by nonlinear registration using FNIRT (FMRIB’s Non-Linear Image Registration Tool), which used a b-spline representation of the registration warp field^60^. The spatially normalized images were then averaged to create a study-specific template, to which the native grey matter images were registered again using both linear and nonlinear algorithms as described above. The registered partial volume images were then modulated by dividing them with the Jacobian of the warp field to correct for local expansion or contraction. The modulated segmented images, which represent the GMV, were then smoothed with an isotropic Gaussian kernel with a 3 mm standard deviation.

The whole process was done with the abovementioned FSL-VBM toolbox. First, a voxel-wise general linear model was built to examine the partial correlation between the resulting grey matter images and SNS addiction scores. Specifically, partial Pearson correlations were estimated in the FSL package, in order to capture the addiction-GMV correlations after accounting for variation explained by the control variables. Seven regressors were included in the model (SNS addiction scores, age, gender, number of contacts on the SNS, SNS use frequency, years of experience with the SNS and whole brain GMV). All continuous variables were, as recommended, mean centered (see (http://mumford.fmripower.org/mean_centering). Non-parametric permutation methods (Randomise v2.1 in FSL; see http://fsl.fmrib.ox.ac.uk/fsl/fsl-4.1.9/randomise/index.html) were used for inference on statistic maps. The null distribution at each voxel was constructed using 10,000 random permutations of the data to ensure that observed results are not due to chance. Because there are hundreds of voxels in the areas of interest, we corrected for multiple comparisons using Threshold-free cluster enhancement (TFCE) with p < 0.05 across the whole brain. Then, to illustrate the correlation, averaged GMV in the significant clusters were extracted.

As a complementary hypothesis driven analysis, GMV of anatomically defined ROIs of five brain regions (bilateral amygdala, bilateral NAc, and MCC) were also extracted. Harvard-Oxford cortical and subcortical probability atlas (25 threshold, 2 mm resolution) was used to extract the anatomical ROIs. The ACC/MCC ROI was defined as the cingulate gyrus, anterior division. Averaged GMV in each ROI was extracted for each participant using the *fslmeants* to sum voxel-by-voxel GMV within the ROI. These total GMV scores were then subjected to partial correlation analyses (accounting for the effects of control variables) using SPSS 24.

## Additional Information

**How to cite this article:** He, Q. *et al*. Brain anatomy alterations associated with Social Networking Site (SNS) addiction. *Sci. Rep.* **7**, 45064; doi: 10.1038/srep45064 (2017).

**Publisher's note:** Springer Nature remains neutral with regard to jurisdictional claims in published maps and institutional affiliations.

## Supplementary Material

Supplementary Information

## Figures and Tables

**Table 1:** Summary of voxel-wise VBM results*.

Brain region	Voxels	Cluster Geometric Center (MNI)			Partial-Correlation with SNS addiction	
		x	y	Z		
Negative correlation between GMV and SNS addiction score (Supports H1)						
R Amygdala	194	23.7	−3.5	−18.0	−0.66 (0.001)	Bilateral Amygdala −0.71 (0.001)
L Amygdala	186	−24.3	−5.6	−17.4	−0.67 (<0.001)	
Positive correlation between GMV and SNS addiction score (Does not support H2)						
ACC/MCC	154	3.3	0.2	36.1	0.65 (<0.001)	

*MNI: Montreal Neurological Institute coordinates; VBM: Voxel-based morphometry; GMV: Grey Matter Volume; ACC/MCC: Anterior/mid cingulate cortex; L: Left; R: Right.
