# Supplementary material for: Brain anatomy alterations associated with Social Networking Site (SNS) addiction
Source: Sci Rep. 2017 Mar 23;7:45064. doi: 10.1038/srep45064 (PMC5362930; doi:10.1038/srep45064)
Supplement: Supplementary Information [file srep45064-s1.pdf]

Supplementary Materials

Brain anatomy alterations associated with Social Networking Site (SNS) addiction

Qinghua He[1,2,**], Ofir Turel[2,3*,**], Antoine Bechara[2]

[1] Faculty of Psychology, Southwest University, Beibei, Chongqing, China
[2] Brain and Creativity Institute, Department of Psychology, University of Southern California, Los Angeles, California, USA
[3] Information Systems and Decision Sciences, California State University, Fullerton, , Fullerton, California, USA

Corresponding author contact information:
800 N. State College Blvd., Fullerton, CA, USA 92834
Phone: +1 (657) 278-5613
Email: oturel@fullerton.edu; ot_739@usc.edu

* Corresponding author
** These authors made equal contributions and can be considered as co-first authors

Appendix A – Neuroscience Glossary

Brain Morphology – Morphology is a term borrowed from biology. It encapsulates a description of the specific structural features of organisms. Applied to the brain, it includes descriptions of the grey matter volumes and content (white matter, CSF, etc.) of specific brain regions, or the whole brain.

Grey Matter – Grey matter includes information processing neuro-components such as neuronal cell bodies, dendrites and axon terminals, as well as fat in the form of glial cells[1,2]. A good analogy for it is a local network of interconnected computing processors which work together on a specific set of tasks. Using this analogy, the neurons process information and communicate via the dendrites attached to them, information to and from other neurons. This structure, since we deal with co-located neurons, resembles this of a local area network, and the processing function of the neurons resembles this of computer processors. The size of this network of neuro-components is captured as grey matter volume (GMV)[3], a measure which we apply in this study.

Amygdala - The amygdala is a pea size structure that exists on each side of the brain (i.e., there is a right and left amygdala). The amygdala is included as a component of the limbic brain system, which is involved in many cue-induced automatic-response processes, such as primary emotions and impulsions[4,5]. For this study, we focus on the amygdala’s role in the development of impulsive decisions, which are characteristics of addictive behaviors. In this context, the amygdala is a key component of the neural system that links conditioned cues in the environment to brain neurotransmitter systems concerned with reward, namely mesolimbic dopamine, and its projection to the nucleus accumbens[6]. Engaging this amygdala system through exposure to conditioned environmental cues elicits automatic and obligatory behaviors, or impulsions[7]. This conditioned cues function seems to play a role in producing negative states in response to expectations of, or actual, exposure to drug cues, especially during abstinence, thus promoting problematic behavior reinstatement[8] Hence, besides the nucleus accumbens, the amygdala is also a key substrate involved in the development and maintenance of addictions[9]. Its hyper-sensitization to cues related to the addiction is one brain process that generates strong impulsions and drives the development of addictions[10].

Nucleus Accumbens (NAc) - This region is part of the ventral striatum and is a key element in people's reward system[11]; it is included in the extended amygdala circuitry[8]. Given its centrality in reward anticipation and processing, it is an important component in decision making[12,13]; when it becomes deficient (overly stimulated) it promotes approach behavior which can be risky[7,14]. For instance, in the classic Olds and Milner study, when it was stimulated in rats it promoted continues stimulation seeking at the expense of basic needs such as eating and drinking[15]. As such, the NAc is a key region in addiction formation and maintenance; it mediates reward assessment which promotes the seeking and repetition of behaviors, even when such behaviors are deemed by society to be problematic[7]. As such, it has been implicated in many substance and non-substance addictions, including to alcohol, cocaine, nicotine, cannabis, opiates and gambling[9,16-19].

Anterior-/Mid- Cingulate Cortex (ACC/MCC) - This area is the front to middle part of the cingulate cortex, which surrounds the frontal part of the corpus callosum[20]. The ACC and adjacent MCC are especially relevant for weak inhibition abilities and consequent addictions[21,22]. Its centrality in behavior inhibition processes stems from several tasks with which the ACC/MCC is involved, including monitoring behavioral conflicts[20], reward anticipation and decision making[23,24], processing feedback[25] and impulse control[26]. All of these tasks are important for inhibiting potent and rewarding behaviors, especially when other alternative behaviors are considered and conflict is created (e.g., should I use the SNS now or study for the exam?)[27]. Specifically, the ACC/MCC circuitry is implicated in the assessment of the salience of expected rewards, assessment of motivational content and assigning emotional valence to behavioral choices, resolving decision conflicts and learning to adjust reward assessments through conditional learning[20-23,28]. Moreover, it has extensive connections with the amygdala and it is therefore involved, using the abovementioned mechanisms, in trying to control the impulsions transmitted via the amygdala[21,27]. Note that we focus in this short description only on tasks related to addiction and low inhibition ability. These tasks, however, also make the ACC/MCC an important substrate involved with many other disorders associated with impaired behavioral control abilities, such as attention deficit-hyperactivity, schizophrenia and obsessive-compulsive disorders[21,27]. Taken together, the ACC/MCC has been linked repeatedly and across numerous studies to the ability to self-control or inhibit impulsive behaviors; and as such, deficient ACC/MCC is often involved with addictions[20,21,28-30].

Voxel Based Morphometry (VBM) – VBM is a neuroimaging technique which allows for assessing the grey matter volumes of different brain regions[31]. It is based on images extracted in structural scans and is advantageous compared with traditional morphometry techniques. This advantage lies in the fact that it registers the grey matter encapsulated in each voxel (and its neighbors; through a smoothing process), as opposed to capturing grey matter in full brain regions, and is hence relatively precise in capturing grey matter volumes even is small brain regions (anatomically predefined collections of voxels), such as the amygdala[32]. Each brain is different, and this technique therefore brings all brains to a common space through a three-dimensional stretching process. Comparisons and grey matter extractions for regions of interest are performed in this common space and the results are then de-transformed to the original space[31]. This technique has gained momentum in neuroscience research[33], in part presumably given its advantages compared with and added value to functional imaging techniques[34]. These include task independence (the results will not change if the task changes, like in functional MRI studies), and the natural environment of measurement (variables of interest, such as addiction or task performance are often measured out of the scanner; and only stable structural features are captured in the scanner).

Appendix B – Measures

Table B1: Survey Items

Variable & Source	Items
SNS addiction [35,36]	How often…. (1=Never, 5=Very Often)<br>- do you find it difficult to stop using Facebook when you are online [or bored]?<br>- do you continue to use Facebook despite your intention to stop?<br>- do others (e.g., parents, siblings, friends) say you should use Facebook less?<br>- do you prefer to use Facebook instead of spending time with others (e.g., family, friends)?<br>- are you short of sleep because of Facebook?<br>- do you think about Facebook even when not online?<br>- do you look forward to your next Facebook session?<br>- do you think you should use Facebook less often?<br>- have you unsuccessfully tried to spend less time on Facebook?<br>- do you rush through your homework or chores in order to use Facebook?<br>- do you neglect your daily obligations (school, chores, or family life) because you prefer to use Facebook?<br>- do you use Facebook when you are feeling down?<br>- do you use Facebook to escape from your sorrows or get relief from negative feelings?<br>- do you feel restless, frustrated, or irritated when you cannot use Facebook?
Age	What is your age? ______ years old
Sex	What is your sex? Female (1)/ Male (0)
Contacts	How many contacts do you have on Facebook? ______ contacts
Use frequency	Considering your average behavior for the previous 4 weeks, how many times per day do you use Facebook? ______ times per day
Years of experience	How long ago did you start using Facebook? ______ years
Medical conditions (Exclusion criteria)	Do you have any of the conditions listed below? (Yes/ No/ I do not Know)<br>- non-corrected bad vision - peripheral vascular disease - diabetes - Reynaud's phenomenon – cryoglobulinemia – vasculitis – lupus - any peripheral neuropathies<br>A computerized short version of the structural clinical interview for DSM-IV Disorders (SCID)<br>- Psychoses, Current major depression episode, A history of major depression episodes or major depressive disorder, Heavy drinking, Substance abuse, Pathological gambling, Schizophrenia, Current and history of anxiety disorders, Bipolar disorder

References:

1 Hansen, M. B., Jespersen, S. N., Leigland, L. A. & Kroenke, C. D. Using diffusion anisotropy to characterize neuronal morphology in gray matter: the orientation distribution of axons

and dendrites in the NeuroMorpho.org database. *Frontiers in integrative neuroscience* **7**, 31-31, doi:10.3389/fnint.2013.00031 (2013).
2 Kassem, M. S. *et al.* Stress-Induced Grey Matter Loss Determined by MRI Is Primarily Due to Loss of Dendrites and Their Synapses. *Molecular Neurobiology* **47**, 645-661, doi:10.1007/s12035-012-8365-7 (2013).
3 Pfefferbaum, A. *et al.* A quantitative magnetic-resonance-imaging study of changes in brain morphology from infancy to late adulthood. *Archives of Neurology* **51**, 874-887 (1994).
4 de Fonseca, F. R. & Navarro, M. Role of the limbic system in dependence on drugs. *Annals of Medicine* **30**, 397-405 (1998).
5 Robbins, T. W. & Everitt, J. Limbic-striatal memory systems and drug addiction. *Neurobiology of Learning and Memory* **78**, 625-636, doi:10.1006/nlme.2002.4103 (2002).
6 Bechara, A., Damasio, H., Damasio, A. R. & Lee, G. P. Different contributions of the human amygdala and ventromedial prefrontal cortex to decision-making. *Journal of Neuroscience* **19**, 5473-5481 (1999).
7 Noel, X., Brevers, D. & Bechara, A. A neurocognitive approach to understanding the neurobiology of addiction. *Current Opinion in Neurobiology* **23**, 632-638 (2013).
8 Koob, G. F. & Volkow, N. D. Neurocircuitry of Addiction. *Neuropsychopharmacology* **35**, 217-238, doi:10.1038/npp.2009.110 (2010).
9 Di Chiara, G. *et al.* Drug addiction as a disorder of associative learning: role of nucleus accumbens shell and extended amygdala dopamine. *Annals of the New York Academy of Science (Advancing from the Ventral Striatum to the Extended Amygdala)* **877**, 461-485 (1999).
10 Everitt, B. J. *et al.* in *Advancing from the ventral striatum to the extended amygdala* Vol. 877 (ed J. F McGinty) 412-438 (Annals of the New York Academy of Science, 1999).
11 Meshi, D., Morawetz, C. & Heekeren, H. R. Nucleus accumbens response to gains in reputation for the self relative to gains for others predicts social media use. *Frontiers in Human Neuroscience* **7**, doi:10.3389/fnhum.2013.00439 (2013).
12 Bechara, A. Decision-making, impulse control, and loss of willpower to resist drugs: A neurocognitive perspective. *Nature Neuroscience* **8**, 1458-1463 (2005).
13 Bechara, A., Dolan, S. & Hindes, A. Decision-making and addiction (part II): myopia for the future or hypersensitivity to reward? *Neuropsychologia* **40**, 1690-1705, doi:10.1016/s0028-3932(02)00016-7 (2002).
14 Brand, M., Young, K. S., Laier, C., Wölfling, K. & Potenza, M. N. Integrating psychological and neurobiological considerations regarding the development and maintenance of specific Internet-use disorders: An Interaction of Person-Affect-Cognition-Execution (I-PACE) model. *Neuroscience & Biobehavioral Reviews* **71**, 252-266, doi:http://dx.doi.org/10.1016/j.neubiorev.2016.08.033 (2016).
15 Olds, J. & Milner, P. Positive reinforcement produced by electrical stimulation of septal area and other regions of rat brain. *Journal of Comparative and Physiological Psychology* **47**, 419-427 (1954).
16 Carelli, R. M. & Wightman, R. M. Functional microcircuitry in the accumbens underlying drug addiction: insights from real-time signaling during behavior. *Current Opinion in Neurobiology* **14**, 763-768, doi:10.1016/j.conb.2004.10.001 (2004).
17 Ernst, M. *et al.* Amygdala and nucleus accumbens in responses to receipt and omission of gains in adults and adolescents. *Neuroimage* **25**, 1279-1291, doi:10.1016/j.neuroimage.2004.12.038 (2005).

18 Gilman, J. M. *et al.* Cannabis Use Is Quantitatively Associated with Nucleus Accumbens and Amygdala Abnormalities in Young Adult Recreational Users. *Journal of Neuroscience* **34**, 5529-5538, doi:10.1523/jneurosci.4745-13.2014 (2014).

19 Quintero, G. C. Role of nucleus accumbens glutamatergic plasticity in drug addiction. *Neuropsychiatric Disease and Treatment* **9**, 1499-1512, doi:10.2147/ndt.s45963 (2013).

20 Bush, G., Luu, P. & Posner, M. I. Cognitive and emotional influences in anterior cingulate cortex. *Trends in Cognitive Sciences* **4**, 215-222, doi:10.1016/S1364-6613(00)01483-2 (2000).

21 Devinsky, O., Morrell, M. J. & Vogt, B. A. Contributions of anterior cingulate cortex to behavior. *Brain* **118**, 279-306, doi:10.1093/brain/118.1.279 (1995).

22 Goldstein, R. Z. *et al.* Role of the anterior cingulate and medial orbitofrontal cortex in processing drug cues in cocaine addiction. *Neuroscience* **144**, 1153-1159, doi:10.1016/j.neuroscience.2006.11.024 (2007).

23 Bush, G. *et al.* Dorsal anterior cingulate cortex: A role in reward-based decision making. *Proceedings of the National Academy of Sciences of the United States of America* **99**, 523-528, doi:10.1073/pnas.012470999 (2002).

24 Williams, Z. M., Bush, G., Rauch, S. L., Cosgrove, G. R. & Eskandar, E. N. Human anterior cingulate neurons and the integration of monetary reward with motor responses. *Nature Neuroscience* **7**, 1370-1375, doi:10.1038/nn1354 (2004).

25 Enriquez-Geppert, S. *et al.* The morphology of midcingulate cortex predicts frontal-midline theta neurofeedback success. *Frontiers in Human Neuroscience* **7**, doi:10.3389/fnhum.2013.00453 (2013).

26 Feja, M. & Koch, M. Ventral medial prefrontal cortex inactivation impairs impulse control but does not affect delay-discounting in rats. *Behavioural Brain Research* **264**, 230-239, doi:10.1016/j.bbr.2014.02.013 (2014).

27 van Veen, V. & Carter, C. S. The anterior cingulate as a conflict monitor: fMRI and ERP studies. *Physiology & Behavior* **77**, 477-482, doi:10.1016/s0031-9384(02)00930-7 (2002).

28 Loh, K. K. & Kanai, R. Higher Media Multi-Tasking Activity Is Associated with Smaller Gray-Matter Density in the Anterior Cingulate Cortex. *Plos One* **9**, doi:10.1371/journal.pone.0106698 (2014).

29 Ansell, E. B., Rando, K., Tuit, K., Guarnaccia, J. & Sinha, R. Cumulative Adversity and Smaller Gray Matter Volume in Medial Prefrontal, Anterior Cingulate, and Insula Regions. *Biological Psychiatry* **72**, 57-64, doi:10.1016/j.biopsych.2011.11.022 (2012).

30 Szeszko, P. R. *et al.* Anterior cingulate grey-matter deficits and cannabis use in first-episode schizophrenia. *British Journal of Psychiatry* **190**, 230-236, doi:10.1192/bjp.bp.106.024521 (2007).

31 Ashburner, J. & Friston, K. Voxel-based morphometry--the methods. *Neuroimage* **11**, 805-821 (2000).

32 Draganski, B. *et al.* Neuroplasticity: Changes in grey matter induced by training - Newly honed juggling skills show up as a transient feature on a brain-imaging scan. *Nature* **427**, 311-312, doi:10.1038/427311a (2004).

33 He, Q. *et al.* Gray and white matter structures in the midcingulate cortex region contribute to body mass index in Chinese young adults. *Brain Struct Funct* **220**, 319-329, doi:10.1007/s00429-013-0657-9 (2015).

34 Kanai, R. & Rees, G. The structural basis of interindividual differences in human behaviour and cognition. *Neuroscience - Nature Reviews* **12**, 231-242 (2011).

35 Meerkerk, G. J., Van Den Eijnden, R. J. J. M., Vermulst, A. A. & Garretsen, H. F. L. The Compulsive Internet Use Scale (CIUS): Some Psychometric Properties. *Cyberpsychology & Behavior* **12**, 1-6, doi:10.1089/cpb.2008.0181 (2009).
36 van Rooij, A. J., Schoenmakers, T. M., Vermulst, A. A., van den Eijnden, R. & van de Mheen, D. Online video game addiction: identification of addicted adolescent gamers. *Addiction* **106**, 205-212, doi:10.1111/j.1360-0443.2010.03104.x (2011).
